# Added value of mindfulness in a digital cannabis intervention: Three-arm randomized trial

**DOI:** 10.1016/j.invent.2026.101021

**Published:** 2026-11-03

**Authors:** Nikolaos Boumparis, Severin Haug, Christian Baumgartner, Andreas Wenger, Doris Malischnig, Michelle Dey, Michael P. Schaub

**Affiliations:** a Swiss Research Institute for Public Health and Addiction, University of Zurich, Switzerland; b Department of Adult Psychiatry and Psychotherapy, Psychiatric University Clinic Zurich, Switzerland; c Therapy Center Ybbs, Vienna Healthcare Group, Vienna, Austria; d Department of Health Science, Institute of Public Health, Zurich University of Applied Sciences, Switzerland

**Keywords:** Cannabis, Digital intervention, Self-help, Mindfulness, Cognitive behavioural therapy, Randomized controlled trial

## Abstract

**Purpose:**

Digital interventions for cannabis use typically produce small short-term effects that fade over time. This three-arm randomized controlled trial examined whether integrating mindfulness into an established digital cognitive behavioural therapy (CBT) self-help intervention improves the durability of outcomes.

**Methods:**

We randomized 301 frequent adult cannabis users to Mindfulness CANreduce (mindfulness-enhanced CBT), Standard CANreduce (CBT only), or a waitlist control group with continued access to care as usual. The primary outcome was self-reported cannabis use days in the past 30 days, assessed at 6 weeks, 3 months, and 6 months, and analysed on an intention-to-treat basis using zero-inflated negative binomial mixed models with multiple imputation.

**Main results:**

Retention was 51.8% at 6 weeks and 31.6% at 6 months, and 6-month attrition did not differ across groups. At 6 weeks, both active interventions reduced cannabis use days relative to the control group (IRR = 0.76, 95% CI [0.64, 0.90]; IRR = 0.80, 95% CI [0.67, 0.95]). At 6 months, the Mindfulness CANreduce group reported fewer use days than both the Standard CANreduce group (IRR = 0.52, 95% CI [0.43, 0.64]) and the control group (IRR = 0.46, 95% CI [0.38, 0.56]), with greater improvements in depressive and anxiety symptoms. Complete-case and tipping-point sensitivity analyses supported these findings, which remain conditional on assumptions about the missing data.

**Conclusion:**

Integrating mindfulness into a fully automated digital intervention was associated with more durable reductions in cannabis use. Given the low retention and engagement, these findings should be interpreted cautiously.

## Highlights

• First RCT of mindfulness-enhanced digital CBT self-help for frequent cannabis use
• Both active programs reduced cannabis use days at 6 weeks versus waitlist control.
• Only the mindfulness version sustained reductions at 6 months (IRR 0.52 vs CBT).
• Depressive and anxiety symptoms also improved more with integrated mindfulness.
• Findings robust in complete-case and tipping-point sensitivity analyses

## 1 Introduction

Cannabis remains one of the most commonly used psychoactive substances worldwide (Wang et al., 2024), with particularly high usage rates among young adults. In Canada, 48% of individuals aged 20–24 report past-year use (Health Canada, 2024), and studies in the United States show comparable rates of around 42% among adults aged 19–30 (Patrick et al., 2024). Recent use is 23% among Australians aged 20–29 years and around 26.5% among New Zealand young adults aged 18–24 years (Ministry of Health, 2024; Australian Institute of Health and Welfare, 2025). In Switzerland, approximately 4% of individuals aged 15 to 64 used cannabis in the past month and 7.6% in the past year (Federal Statistical Office (FSO), 2022).

Among those entering addiction treatment for illegal drug use in Switzerland in 2023, cannabis was the primary problem for 38.8% (Krizic et al., 2024). In Europe, around 92,000 people sought specialized treatment for cannabis-related disorders in 2022, accounting for 36% of all substance abuse treatment requests (European Union Drugs Agency, 2024). Treatment demand may grow as regulatory frameworks evolve. In Switzerland, legislative amendments in March 2021 permit scientific pilot studies on the regulated distribution of cannabis for recreational use (Cattacin, 2021). This reflects broader international trends towards cannabis legalization and regulation (Bahji and Stephenson, 2019). These policy shifts may alter patterns of use, underscoring the importance of accessible, evidence-based interventions (National Academies of Sciences, Engineering, and Medicine, 2024).

Frequent cannabis consumption is associated with an elevated risk of developing Cannabis Use Disorder (CUD) (Leung et al., 2020), commonly co-occurs with mood and anxiety disorders (Hall and Degenhardt, 2009; Onaemo et al., 2021), and is linked to adverse physical, social, and occupational outcomes (Hall, 2015). Despite these risks, less than 8% of individuals with CUD seek professional help (Choi et al., 2024). Commonly reported barriers include stigma, financial cost, limited geographic access, a preference for self-management, scheduling conflicts, and concerns about abstinence requirements (van der Pol et al., 2013).

In response to these barriers, digital interventions have emerged as accessible alternatives, offering anonymity and flexible, self-directed formats (Monarque et al., 2023). Meta-analyses suggest that digital interventions grounded in Cognitive Behavioural Therapy (CBT) and Motivational Interviewing (MI) yield small to moderate short-term reductions in cannabis use (Côté et al., 2024; Boumparis et al., 2019; Hoch et al., 2016). The CANreduce programme, a self-help digital intervention incorporating MI and CBT elements, has demonstrated efficacy relative to waitlist controls (WLCs), particularly when enhanced with automated guidance to improve adherence (Schaub et al., 2015; Baumgartner et al., 2021): in the CANreduce 2.0 trial, adherence-focused guidance reduced cannabis use days relative to internet as usual with d = 0.60 (Baumgartner et al., 2021), exceeding the pooled short-term effects reported in meta-analyses (g = 0.11–0.16) (Boumparis et al., 2019; Hoch et al., 2016; Tait et al., 2013). These effects still leave room for refinement, particularly in user engagement, personalization, and sustaining effects beyond 6–12 months.

Mindfulness-based interventions (MBIs) represent a promising adjunctive or alternative approach for individuals with substance use disorders (SUDs). Mindfulness, defined as purposeful, non-judgmental attention to present-moment experiences (Kabat-Zinn, 2003), operates through several mechanisms relevant to recovery, including increased metacognitive awareness of triggers and habitual responses, acceptance of distressing internal states, reduced cue reactivity through decentering, and enhanced emotion regulation capacity (Garland and Howard, 2018; Witkiewitz et al., 2013). Consistent with these mechanisms, momentary mindfulness following a digitally supported intervention for frequent cannabis use has been associated with lower negative affect and reduced cannabis desire (Shrier and Harris, 2023). Meta-analyses indicate that in-person MBIs have small effects on substance use, moderate effects on craving, and large effects on stress (Li et al., 2017), and large effects on craving and negative affectivity relative to active comparators (Cavicchioli et al., 2018). However, studies specifically targeting frequent cannabis users remain limited and primarily small-scale (Dakwar and Levin, 2013).

Although MBIs have demonstrated potential, their accessibility is often limited by the same barriers that constrain traditional care. Structured mindfulness practices, such as guided meditations and body scans, lend themselves well to digital delivery, which may broaden access and allows standardized delivery and repeated practice.

A central unresolved problem in this field, however, is the durability of treatment effects: pooled short-term effects of digital cannabis interventions are small and were no longer significant at follow-up in a previous meta-analysis (Boumparis et al., 2019). To address this gap, the current study evaluated CANreduce 3.0, to our knowledge the first randomized controlled trial comparing a self-help digital intervention integrating mindfulness and CBT with both an active comparator (CBT adapted from CANreduce 2.0) and a WLC over 6 months. The aim was to determine whether integrating mindfulness (Mindfulness CANreduce) improves long-term cannabis use and mental health outcomes compared to CBT alone (Standard CANreduce) and a WLC.

## 2 Methods

### 2.1 Study design

This three-arm, parallel-group randomized controlled trial (RCT) compared a CANreduce intervention combining mindfulness and cognitive behavioural therapy (CBT) (Arm 1) with a standard CBT-only CANreduce as active comparator (Arm 2) and a waitlist control (WLC) (Arm 3) in frequent adult cannabis users. The digital interventions were developed and are reported in accordance with the Template for Intervention Description and Replication (TIDieR) checklist. Given the low anticipated risk based on previous trials and continuous safety monitoring throughout the study, no interim analyses or formal stopping guidelines were planned.

### 2.2 Trial registration and protocol

The full study protocol has been published (Dey et al., 2022). The trial was registered with the International Standard Randomized Controlled Trial Number (ISRCTN) Registry on September 9, 2021 (trial number: ISRCTN14971662; URL: https://www.isrctn.com/ISRCTN14971662).

### 2.3 Ethical approval and consent to participate

The study protocol was approved by the Ethics Committee of the Faculty of Arts and Social Science at the University of Zurich, Switzerland (approval number: 20.4.17), and the study was conducted in accordance with the Declaration of Helsinki. All participants provided digital informed consent before starting the intervention.

### 2.4 Participants

Participants were recruited from the general population between September 2021 and January 2024 via study websites (canreduce.ch, canreduce.at). Recruitment was promoted through online advertisements, newspaper articles, addiction help portals, and materials for healthcare professionals.

Inclusion criteria were: (1) Age ≥ 18 years; (2) Cannabis use at least weekly in the past 30 days; (3) Weekly internet access with capability for undisturbed audio/video playback; (4) A valid email address and phone number; (5) German fluency sufficient for study materials; (6) Provided informed consent.

Exclusion criteria were: (1) Current treatment for cannabis use; (2) Current pharmacologically treated psychiatric disorder, or a history of psychosis, schizophrenia, or bipolar I disorder; (3) Current suicidal ideation on the P4 Suicidality Screener (Dube et al., 2010), as assessed at screening. Individuals receiving pharmacological treatment for a psychiatric disorder were excluded to prevent confounding from concurrent treatments and because the fully automated programme provides no clinical oversight. Participation in earlier CANreduce trials was not an exclusion criterion and was not systematically assessed.

### 2.5 Procedure

A study website informed interested individuals about study aims, procedures, randomization, data handling, and participant rights. After giving informed consent, participants registered with a username, email, and phone number. An online screening questionnaire then assessed eligibility. Eligible participants completed the online baseline assessment (t0) and were automatically randomized to one of three study arms.

Participants in the Mindfulness CANreduce (Arm 1) and Standard CANreduce (Arm 2) arms received immediate access to their digital interventions.

Follow-up assessments occurred at 6 weeks (t1, end of intervention for Arms 1–2), 3 months (t2), and 6 months (t3). Participants received automated email reminders for each follow-up assessment. In addition, a member of the study team contacted participants by telephone and offered to complete the assessment over the phone. A participant's decision not to complete an assessment was respected. Compensation for follow-up assessments (CHF 20 for t2, CHF 30 for t3) was provided as an online voucher or charitable donation. No compensation was offered for the 6-week assessment (t1); the compensation budget was deliberately concentrated on the later follow-ups, where attrition risk was expected to be greatest.

Participant blinding to allocation (active vs. WLC) was not possible. However, participants in the active arms (1 and 2) were unaware of specific intervention differences. Data analysts were not blinded.

Individuals reporting any current suicidal ideation were excluded at screening, and an instant help page, accessible at all times, provided instructions and emergency contacts.

### 2.6 Randomization

Following baseline assessment, participants were randomized to the three study arms in a 1:1:1 ratio using simple randomization without blocking or stratification, via an automated computer-generated sequence embedded in the server backend. Allocation was concealed because the sequence was inaccessible to study personnel and was revealed to participants only after the completion of the baseline assessment. As enrollment and randomization were fully automated, no study personnel were involved in assigning participants to interventions.

### 2.7 Interventions

The CANreduce interventions were developed iteratively, with initial versions informed by focus groups with the target population, and both current interventions were built upon this work. There was no direct patient involvement in the development of the new mindfulness-based components for CANreduce 3.0, but an external certified mindfulness trainer was consulted to ensure the quality and appropriateness of the content.

Both active interventions were delivered over 6 weeks via the CANreduce 3.0 web platform (computer, tablet, or smartphone). Each included eight modules, a dashboard, consumption diary, activity planner, fictional peers, and guidance from an avatar eCoach (Dey et al., 2022).

Mindfulness CANreduce (Arm 1) combined CBT principles with mindfulness practices adapted from Mindfulness-Based Relapse Prevention (Bowen et al., 2009). Its eight modules addressed motivation, triggers, craving, and relapse prevention, each enriched with mindfulness concepts and formal practices adapted from MBRP, including mindful breathing, body scanning, urge surfing, the SOBER breathing space, mountain and walking meditation, lovingkindness and self-compassion, and recognizing thoughts as thoughts. Practices were introduced in written form, explicitly linked to cannabis reduction, and delivered as audio files of varying lengths; they remained accessible throughout and after the 6-week programme. The eCoach provided text-based summaries and guided audio exercises. Participants tracked cannabis use and mindfulness practice in their diary.

Standard CANreduce (Arm 2), the active comparator, was an adapted version of CANreduce 2.0 using only CBT and MI techniques (Baumgartner et al., 2021). It had eight modules with similar content areas to Arm 1 but no mindfulness or audio practices. eCoach guidance was text-only. Text lengths were comparable across active arms.

The WLC group (Arm 3) completed all assessments at the same time points. After randomization, they received brief feedback on cannabis risks and a webpage with harm reduction strategies and emergency contacts but no access to CANreduce modules. They were offered access to the Mindfulness CANreduce intervention after the final t3 assessment.

### 2.8 Assessments

All assessments were self-reported and administered online via the CANreduce 3.0 platform at four time points: baseline (t0), 6 weeks (t1), 3 months (t2), and 6 months (t3). All measures, including the eligibility criterion of at least weekly cannabis use, were self-reported; use was not biochemically verified.

### 2.9 Primary outcome

The primary outcome was the number of self-reported cannabis use days in the preceding 30 days, measured at all four assessment points (t0, t1, t2, and t3).

### 2.10 Secondary outcomes

Cannabis-related secondary outcomes were assessed at all four time points. These included quantity and frequency of use over the past 7 days, assessed via an online-adapted Timeline Follow-Back (TLFB) method (Robinson et al., 2014). Quantity was recorded in individually calibrated standard joints, which participants defined by cannabis type, tobacco admixture, and amount with the help of scaled photographs (see Supplementary Material). This procedure references the quantities of cannabis that each participant customarily uses, rather than assuming a constant joint size. However, it primarily quantifies smoked cannabis. Other routes of administration, such as edibles or disposable vapes, or combinations of methods, are not fully accounted. Additional cannabis-related measures included problematic use patterns, assessed with the Cannabis Use Disorders Identification Test-Revised (CUDIT-R) (Adamson et al., 2010), and dependence severity, evaluated through the Severity of Dependence Scale (SDS) (Gossop et al., 1995).

Psychological outcomes included the Perceived Stress Scale-10 (PSS-10) (Harris et al., 2023) and the Mindful Attention Awareness Scale (MAAS) (Van Dam et al., 2010), which were administered at all four time points. Depressive symptoms, measured by the Patient Health Questionnaire-9 (PHQ-9) (Kroenke et al., 2001), and anxiety symptoms, measured by the Generalized Anxiety Disorder-7 (GAD-7) (Spitzer et al., 2006), were assessed at baseline, 3 months, and 6 months (t0, t2, t3).

Other substance use was evaluated at baseline, 3 months, and 6 months (t0, t2, t3). This included alcohol use, evaluated by the Alcohol Use Disorders Identification Test-Consumption (AUDIT-C) (Simon et al., 2024), and use of other substances, assessed with items from the NIDA-modified ASSIST pre-screener (Oga et al., 2020).

### 2.11 Additional measures

Intervention-related measures were collected from the active arms (1 and 2). Client satisfaction was measured at 6 weeks (t1) with the Client Satisfaction Questionnaire for Internet Interventions (CSQ-I) (Boß et al., 2016). Intervention-attributed negative effects (Rozental et al., 2019) were also assessed at t1. Concurrent treatment utilization was assessed at 3 and 6 months (t2, t3), where participants reported (‘yes’/‘no’) use of other services (e.g., online counseling, drug counseling, general practitioners) besides CANreduce.

Baseline (t0) measures included sociodemographics, cannabis use history, meditation experience, the Adult ADHD Self-Report Scale (ASRS-V1.1) (Silverstein et al., 2018), the Drug Abuse Screening Test (DAST-10) (Villalobos-Gallegos et al., 2015), and the Comprehensive Inventory of Mindfulness Experiences (CHIME) (Bergomi et al., 2015).

### 2.12 Sample size calculation

Power analyses detailed in the study protocol (Dey et al., 2022), simulating data from a prior trial (Baumgartner et al., 2021) to detect a small effect size (Arm 1 vs. Arm 3), indicated a target sample of N = 630 (n = 210 per arm) was needed to achieve >80% power (α = 0.05, two-tailed).

Recruitment proceeded more slowly than anticipated, and enrolment was closed at N = 301 (48% of the planned sample) after a formal assessment of recruitment feasibility.

### 2.13 Statistical analyses

Analyses followed the intention-to-treat (ITT) principle, including all randomized participants in their allocated arm. To address missing data, we utilized Multiple Imputation by Chained Equations (MICE) with the mice package (van Buuren and Groothuis-Oudshoorn, 2011) in R (v. 4.4.1) (R Core Team, 2024). We generated 20 imputed datasets per arm using the Classification and Regression Trees algorithm. Imputation was conducted under the Missing At Random (MAR) assumption, which cannot be verified empirically. The imputation model included baseline demographics and baseline values of all outcome measures as predictors, so that baseline characteristics associated with dropout informed the imputed values.

For the primary outcome (days of cannabis use in the past 30 days), we fitted a Generalized Linear Mixed Model (GLMM) with a Zero-Inflated Negative Binomial (ZINB) distribution, using the glmmTMB package (Brooks et al., 2017), to account for the count nature of the data and the excess zeros. The secondary count outcome (quantity of standard joints) was similarly analysed using GLMMs (ZINB) fitted to rounded imputed values. Secondary continuous outcomes (including CUDIT-R, SDS, PHQ-9, GAD-7, PSS-10, MAAS and AUDIT-C) were analysed using linear mixed models (LMMs) via the lmerTest package (Kuznetsova et al., 2017). All models included fixed effects for time, group, and their interaction, with baseline values as covariates and participant ID as the random intercept.

Engagement was quantified utilizing server logs. Adherence was operationalized as the mean completion rate across all eight modules (proportion of unique pages viewed), and retention as the mean completion rate of the consumption diary across the six intervention weeks. Engagement logs were available only for participants registered on the Swiss platform (canreduce.ch; 174 of the 203 active-arm participants), to whom engagement analyses are restricted. Given the skewed distributions, medians and interquartile ranges (IQRs) are reported alongside means, and group comparisons used Mann-Whitney *U* tests. Client satisfaction (CSQ-I) was compared between active arms using an independent samples *t*-test or a Mann-Whitney *U* test. For negative effects, we calculated frequencies and categorized the change in severity (“Improved,” “No Change,” “Worsened”) based on participant descriptions and Likert scale ratings. This categorization compared the rated severity of each event at occurrence with that at the time of reporting; the small number of reported events prevented more detailed analyses.

All statistical tests were two-tailed with a significance threshold of α = 0.05. For the GLMMs, simulated residual diagnostics using the DHARMa package (Hartig, 2016) confirmed no significant deviation from the expected distribution (Kolmogorov-Smirnov test p > 0.05). For linear mixed models, residual normality was assessed visually and through Shapiro-Wilk tests on model residuals using the performance package (Lüdecke et al., 2021). When normality assumptions were violated (as with the Client Satisfaction Questionnaire), the Mann-Whitney *U* test was used.

The primary hypothesis tested the significance of the Time × Group interaction. The primary comparisons were each active arm versus the WLC on the primary outcome at each follow-up; the comparison between the two active arms addressed the secondary question of the added value of the mindfulness components. Post-hoc pairwise comparisons were conducted using estimated marginal means (EMMs) via the emmeans package (Lenth, 2022), with p-values corrected for the three group comparisons at each time point using Tukey's honestly significant difference (HSD) method. Baseline characteristics of completers and non-completers were compared with Wilcoxon rank-sum tests and Fisher's exact tests. Sensitivity analyses were performed on non-imputed data to assess the robustness of the primary findings. An additional exploratory analysis regressed observed cannabis use days at 6 months on module completion (per 10 percentage points), baseline use, and study arm, using a negative binomial model. In addition, a delta-adjusted (tipping-point) sensitivity analysis probed the MAR assumption for the 6-month primary endpoint by adding increasing values of δ (0–17 days) to the imputed cannabis use days of Mindfulness CANreduce participants with missing 6-month data and refitting the primary model (see Supplementary Material).

## 3 Results

### 3.1 Participant flow and baseline characteristics

The flow of participants through the trial is detailed in the CONSORT diagram (see Fig. 1). Of 524 individuals assessed for eligibility, 223 (42.6%) were excluded, for reasons such as a suicidal ideation score above zero (n = 139, 26.5%), receiving psychiatric medication (n = 90, 17.2%), being in other treatment for cannabis use (n = 35, 6.7%), or not meeting the required cannabis consumption frequency (n = 28, 5.3%); participants could be excluded for multiple reasons. Consequently, 301 (57.4%) participants were randomized: 109 to the Mindfulness CANreduce group (Arm 1), 94 to the Standard CANreduce group (Arm 2), and 98 to the WLC condition (Arm 3).

**Fig. 1:**
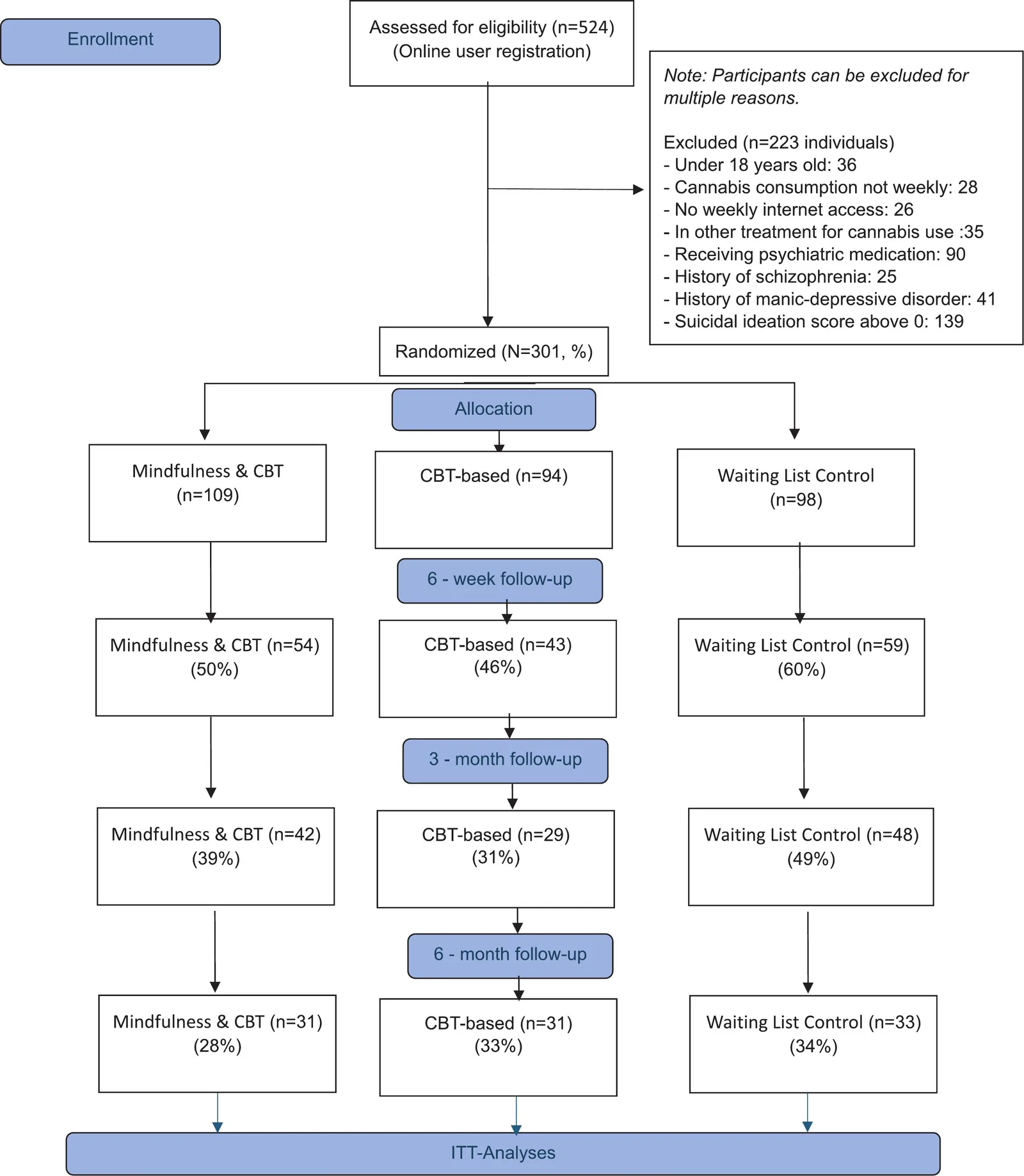
CONSORT diagram.

At the 6-week follow-up, 54 (50%) participants in the Mindfulness CANreduce group, 43 (46%) in the Standard CANreduce group, and 59 (60%) in the WLC group completed the assessment. Completion was 42 (39%), 29 (31%), and 48 (49%) at 3 months and 31 (28%), 31 (33%), and 33 (34%) at 6 months, an overall 6-month completion rate of 31.6%.

Baseline characteristics of randomized participants are displayed in Table 1. The sample was predominantly male (57.8%), with a mean of 26.0 (SD = 6.3) cannabis use days in the past 30 days. Age, recorded at screening for the 257 participants on the Swiss platform, averaged 31.1 years (SD = 8.7, range 18–60) and was comparable across groups. Participants reported high problematic use (CUDIT M = 25.2), moderate dependence (SDS M = 9.2), moderate depressive symptoms (PHQ-9 M = 11.3), and mild-to-moderate anxiety (GAD-7 M = 8.9), and baseline characteristics were comparable across groups.

**Table 1:** Baseline participant data.

Characteristics	Mindfulness N = 109	CBT N = 94	Control N = 98
**Gender, n**			
Female	34 (31.2%)	41 (43.6%)	46 (46.9%)
Male	73 (67.0%)	51 (54.3%)	50 (51.0%)
Diverse	2 (1.8%)	2 (2.1%)	2 (2.0%)
**Age, years, mean (SD)**^a^	31.6 (9.4)	30.6 (8.7)	31.0 (7.9)
**Nationality**			
Swiss	52 (47.7%)	39 (41.5%)	45 (45.9%)
German	36 (33.0%)	33 (35.1%)	41 (41.8%)
Austrian	13 (11.9%)	14 (14.9%)	8 (8.2%)
Italian	4 (3.7%)	1 (1.1%)	2 (2.0%)
Other	4 (3.7%)	7 (7.4%)	2 (2.0%)
**Education**			
Compulsory school	3 (2.8%)	14 (14.9%)	7 (7.1%)
Vocational apprenticeship	32 (29.4%)	22 (23.4%)	24 (24.5%)
Secondary school	21 (19.3%)	23 (24.5%)	14 (14.3%)
Higher vocational training	18 (16.5%)	16 (17.0%)	23 (23.5%)
University	34 (31.2%)	18 (19.1%)	28 (28.6%)
Not specified	1 (0.9%)	1 (1.1%)	2 (2.0%)
**Meditation experience**			
Yes, I have already tried it	37 (33.9%)	30 (31.9%)	42 (42.9%)
Yes, I meditate from time to time	21 (19.3%)	21 (22.3%)	18 (18.4%)
Yes, I meditate regularly (at least once a week)	15 (13.8%)	7 (7.4%)	9 (9.2%)
No experience	36 (33.0%)	36 (38.3%)	29 (29.6%)
**Outcomes, mean (SD)**			
PHQ-9	10.68 (5.14)	11.78 (4.95)	11.44 (5.74)
PSS-10	21.36 (3.38)	21.65 (4.17)	21.35 (4.47)
GAD-7	8.65 (4.96)	9.16 (4.76)	8.85 (5.03)
SDS	9.37 (3.22)	9.14 (3.23)	9.02 (3.27)
Cannabis use past 30 days	25.02 (7.59)	26.60 (5.55)	26.59 (5.33)
Standard joints past 7 days	21.34 (15.36)	22.38 (14.33)	23.78 (14.38)
MAAS	54.28 (12.80)	55.62 (12.40)	56.12 (13.99)
AUDIT-C	3.15 (2.28)	3.05 (2.56)	3.16 (2.42)
CUDIT	25.09 (6.16)	25.91 (6.45)	24.77 (6.64)
DAST-10	4.11 (2.29)	4.24 (2.15)	3.65 (2.48)
ASRS-v1.1	2.72 (1.65)	2.94 (1.73)	2.78 (1.84)
**CHIME subscales**			
Awareness Internal	4.06 (1.04)	4.20 (0.82)	4.19 (0.91)
Awareness External	4.23 (1.15)	4.48 (0.85)	4.29 (1.08)
Acting with Awareness	3.51 (0.89)	3.57 (0.93)	3.51 (0.94)
Accepting/Nonjudgmental	3.06 (1.18)	3.12 (1.18)	3.05 (1.07)
Decentering/Nonreactivity	3.21 (0.97)	3.30 (1.03)	3.16 (0.89)
Openness to Experiences	3.09 (0.90)	3.02 (0.97)	3.20 (0.92)
Relativity of Thoughts	3.84 (0.89)	3.82 (0.95)	3.81 (0.96)
Insightful Understanding	3.76 (0.80)	3.77 (0.88)	3.76 (0.80)

ASRS-v1.1, Adult ADHD Self-Report Scale; AUDIT-C, Alcohol Use Disorders Identification Test; CHIME, Comprehensive Inventory of Mindfulness Experiences; CUDIT, Cannabis Use Disorder Identification Test; DAST-10, Drug Abuse Screening Test; GAD-7, Generalized Anxiety Disorder; MAAS, Mindful Attention Awareness Scale; PHQ-9, Patient Health Questionnaire; PSS-10, Perceived Stress Scale; SDS, Severity of Dependence Scale.

a Age was recorded at screening on the Swiss platform only (Mindfulness n = 94, CBT n = 80, Control n = 83) and is not available for participants on the Austrian platform.

### 3.2 Dropout analysis

Of 301 participants, 156 (51.8%) completed the 6-week post-test and 95 (31.6%) the 6-month follow-up. Attrition did not differ significantly between arms at 6 weeks (χ^2^(2) = 4.38, p = 0.11), or 6 months (χ^2^(2) = 0.78, p = 0.68) but did at 3 months (χ2(2) = 6.67, p = 0.036), when completion was lowest in the Standard CANreduce arm (31%, vs 39% and 49%). Non-completers included significantly more men than completers at 6 weeks (p = 0.027) and 6 months (p = 0.033). No other baseline characteristic differed between completers and non-completers.

### 3.3 Primary outcome: days of cannabis use over the preceding 30 days

Analysis of the primary outcome (days of cannabis use) using a zero-inflated negative binomial GLMM revealed a significant time × group interaction (p < 0.001; see Table 3). Fig. 2 shows the estimated marginal means and the pairwise comparisons. At baseline, groups did not differ significantly (all p-values for pairwise comparisons >0.82).

**Table 3:** Model parameters for primary outcome.

Category	Parameter	β (95% CI)	z	p-value
Group effects	CBT vs Mindfulness	0.04 [−0.10, 0.17]	0.55	0.583
	WLC vs Mindfulness	0.04 [−0.09, 0.18]	0.59	0.554
Time effects	Week 6	−0.33 [−0.47, −0.19]	−4.73	<0.001 ***
	FU1	−0.41 [−0.55, −0.27]	−5.68	<0.001 ***
	FU2	−0.83 [−0.98, −0.68]	−10.95	<0.001 ***
Interaction	CBT × Week 6	0.01 [−0.19, 0.21]	0.11	0.913
	WLC × Week 6	0.24 [0.04, 0.43]	2.40	0.017 *
	CBT × FU1	0.16 [−0.04, 0.36]	1.56	0.120
	WLC × FU1	0.27 [0.08, 0.47]	2.75	0.006 **
	CBT × FU2	0.61 [0.39, 0.82]	5.60	<0.001 ***
	WLC × FU2	0.73 [0.53, 0.94]	7.16	<0.001***

β coefficients are presented on the log scale; exponentiated β values yield IRR. * p < .05, ** p < .01, *** p < .001 (two-tailed).

**Fig. 2:**
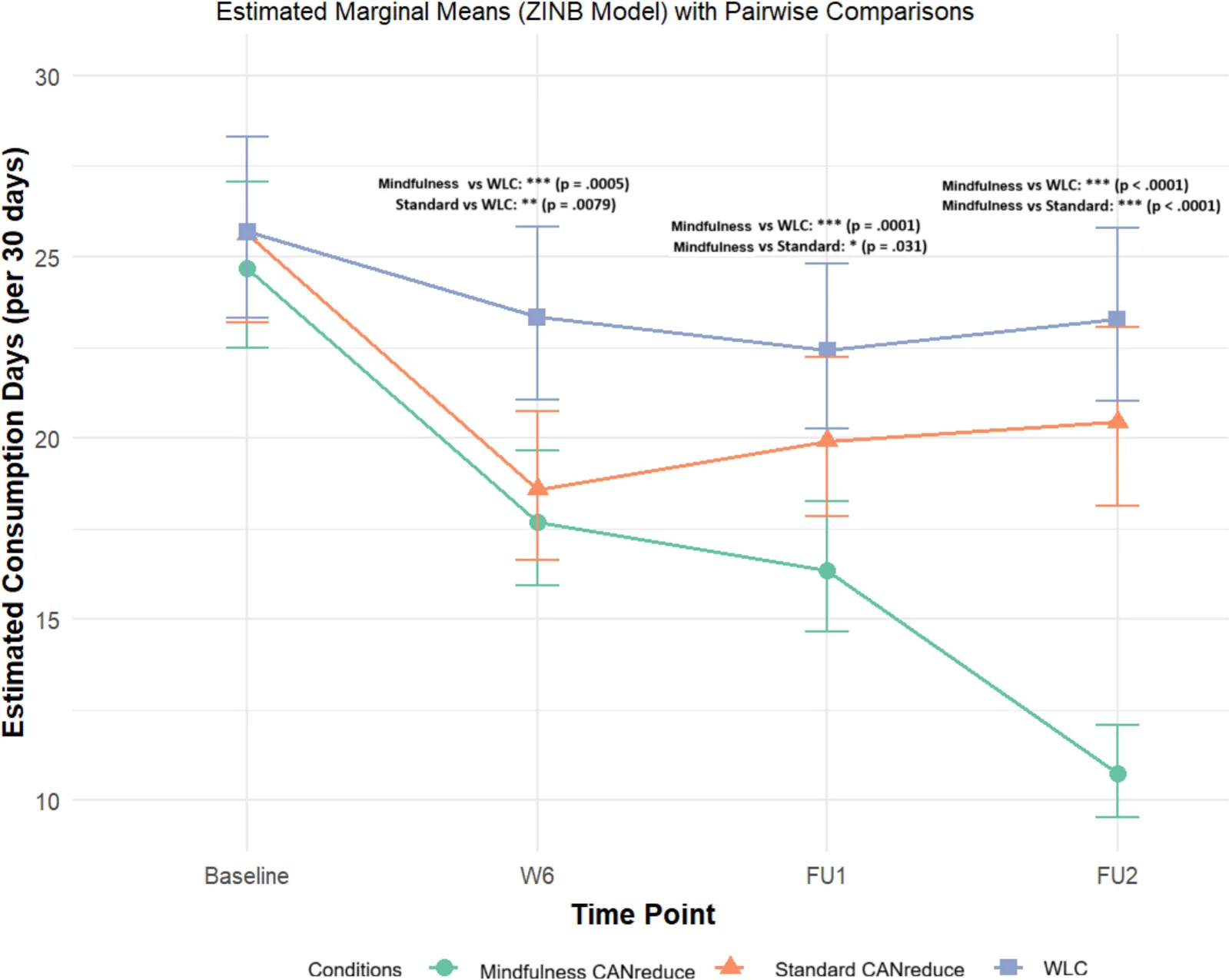
Estimated marginal means of cannabis consumption days by intervention group over time.

At the 6-week post-intervention assessment, both active interventions showed significantly fewer use days than the WLC group (IRR = 0.76, 95% CI [0.64, 0.90], p = 0.001 and IRR = 0.80, 95% CI [0.67, 0.95], p = 0.008, respectively). The two active interventions did not differ significantly from each other (p = 0.80).

At the 3-month follow-up, the Mindfulness CANreduce group was still performing better than the WLC group (IRR = 0.73, 95% CI [0.61, 0.87], p < 0.001), and reported significantly fewer cannabis use days than the Standard group (IRR = 0.82, 95% CI [0.68, 0.99], p = 0.031). The Standard CANreduce group no longer differed significantly from the WLC (p = 0.26).

At the primary endpoint of 6 months (see Table 4 and Fig. 2), participants in the Mindfulness group reported significantly lower consumption than those in the Standard group (IRR = 0.52, 95% CI [0.43, 0.64], p < 0.001) and the WLC group (IRR = 0.46, 95% CI [0.38, 0.56], p < 0.001). There was no significant difference between the Standard and WLC groups (IRR = 0.88, 95% CI [0.73, 1.06], p = 0.24). Within-group analyses confirmed that only the Mindfulness CANreduce group showed a continued significant reduction between 3 and 6 months (p < 0.001).

**Table 4:** Estimated differences between groups at follow-up 2.

Outcome^a^	Comparison	Estimate (IRR)	Std. Error (SE)	95% CI	p-value
**Primary outcome**					
Cannabis use past 30 days	Mindf. vs CBT	0.52	0.045	[0.43, 0.64]	<0.001 ***
	Mindf. vs WLC	0.46	0.037	[0.38, 0.56]	<0.001 ***
	CBT vs WLC	0.88	0.071	[0.73, 1.06]	0.2441
**Secondary outcomes**					
Standard joints past 7 days	Mindf. vs CBT	0.70	0.09	[0.51, 0.96]	0.0224 *
	Mindf. vs WLC	0.47	0.06	[0.35, 0.63]	<0.0001 ***
	CBT vs WLC	0.67	0.09	[0.50, 0.91]	0.0049 **
		**Estimate (mean difference)**			
CUDIT	Mindf. vs CBT	−1.13	0.95	[−3.37, 1.11]	0.4610
	Mindf. vs WLC	−7.40	0.94	[−9.61, −5.18]	<0.0001 ***
	CBT vs WLC	−6.26	0.98	[−8.56, −3.97]	<0.0001 ***
SDS	Mindf. vs CBT	−0.783	0.43	[−1.79, 0.23]	0.1642
	Mindf. vs WLC	−2.180	0.43	[−3.179, −1.180]	<0.0001 ***
	CBT vs WLC	−1.397	0.44	[−2.433, −0.361]	0.0045 **
PHQ-9	Mindf. vs CBT	−1.49	0.58	[−2.87, −0.12]	0.0288 *
	Mindf. vs WLC	−2.70	0.58	[−4.05, −1.34]	<0.0001 ***
	CBT vs WLC	−1.20	0.60	[−2.61, 0.21]	0.1114
GAD-7	Mindf. vs CBT	−1.61	0.50	[−2.78, −0.45]	0.0034 **
	Mindf. vs WLC	−2.20	0.49	[−3.35, −1.05]	<0.0001 ***
	CBT vs WLC	−0.59	0.51	[−1.78, 0.60]	0.4780
AUDIT-C	Mindf. vs CBT	0.21	0.22	[−0.31, 0.73]	0.6080
	Mindf. vs WLC	0.57	0.22	[0.060, 1.09]	0.0246 *
	CBT vs WLC	0.36	0.23	[−0.17, 0.90]	0.2486
PSS	Mindf. vs CBT	−0.25	0.52	[−1.46, 0.97]	0.8810
	Mindf. vs WLC	−2.17	0.51	[−3.37, −0.97]	0.0001 ***
	CBT vs WLC	−1.93	0.53	[−3.17, −0.68]	0.0009 ***
MAAS	Mindf. vs CBT	−0.190	1.29	[−3.22, 2.83]	0.9881
	Mindf. vs WLC	6.287	1.28	[3.30, 9.28]	<0.0001 ***
	CBT vs WLC	6.477	1.32	[3.38, 9.58]	<0.0001 ***

a Incidence rate ratios (IRR) come from zero-inflated negative binomial mixed models; mean differences come from linear mixed models. All models included group, time, their interaction, the baseline value of the outcome as covariate, and a random intercept per participant; contrasts are Tukey-adjusted. Mindf., Mindfulness CANreduce; CBT, Standard CANreduce; WLC, Waitlist Control; CUDIT, Cannabis Use Disorder Identification Test; SDS, Severity of Dependence Scale; PHQ-9, Patient Health Questionnaire-9; GAD-7, Generalized Anxiety Disorder-7; AUDIT-C, Alcohol Use Disorders Identification Test-Consumption; PSS, Perceived Stress Scale; MAAS, Mindful Attention Awareness Scale. * p < .05, ** p < .01, *** p < .001 (two-tailed).

A sensitivity analysis on non-imputed data yielded largely consistent results, with one exception at the 3-month follow-up. Unlike in the imputed analysis, the Standard CANreduce group maintained a significant reduction in cannabis use days compared to the WLC group (IRR = 0.74, 95% CI [0.60, 0.92], p = 0.004), and the two active interventions did not differ significantly (p = 0.98). At six months, the Mindfulness CANreduce intervention was again significantly more effective than the Standard CANreduce intervention (IRR = 0.69, 95% CI [0.53, 0.90], p = 0.003) and the WLC group (IRR = 0.58, 95% CI [0.45, 0.74], p < 0.001), while the Standard CANreduce group did not differ from the WLC group (p = 0.21).

The delta-adjusted tipping-point analysis indicated that the 6-month findings were robust to substantial departures from MAR. The Mindfulness versus Standard CANreduce contrast remained statistically significant unless participants with missing 6-month data in the Mindfulness arm were assumed to use cannabis on average 12 or more days per month above their MAR-based imputed values (a mean of 18.9 use days, against their own baseline mean of 25.6 days), with comparator-arm dropouts following MAR. The Mindfulness versus WLC contrast remained significant unless this departure reached 16 days (a mean of 22.2 use days; see Supplementary Material).

### 3.4 Secondary outcomes

For cannabis quantity (standard joints), a significant Group × Time interaction was found (p < 0.001; Table 2, Table 4). At 6 months, Mindfulness CANreduce showed lower consumption than both Standard CANreduce (IRR = 0.70, p = 0.022) and WLC (IRR = 0.47, p < 0.001), while Standard CANreduce was also superior to WLC (IRR = 0.67, p = 0.005).

**Table 2:** Intention-to-treat outcomes.

Group and measure	Posttreatment mean (SD)	3 month follow-up mean (SD)	6 month follow-up mean (SD)
**Mindfulness-based self-help**			
Cannabis use past 30 days	15.7 (11.5)	13.7 (11.2)	9.41 (10.5)
Standard joints past 7 days	8.99 (10.2)	8.38 (10.4)	6.24 (8.75)
CUDIT	24.4 (7.08)	16.2 (9.15)	13.3 (9.03)
SDS	7.56 (3.71)	6.00 (3.76)	4.97 (3.62)
PHQ-9	N/A	8.58 (5.09)	6.71 (4.73)
GAD-7	N/A	6.52 (4.07)	4.77 (3.27)
AUDIT-C	N/A	2.82 (1.78)	3.27 (2.26)
PSS	21.0 (3.06)	20.2 (4.65)	18.5 (4.80)
MAAS	55.4 (12.8)	60.1 (13.7)	63.0 (14.3)
CSQ-I	27.0 (4.72)	N/A	N/A
**CBT-based self-help**			
Cannabis use past 30 days	17.2 (10.6)	18.1 (11.3)	14.9 (12.2)
Standard joints past 7 days	11.2 (11.5)	11.4 (12.8)	8.63 (9.79)
CUDIT	22.1 (7.22)	17.7 (8.78)	15.0 (9.06)
SDS	6.99 (2.52)	6.76 (2.53)	5.63 (4.36)
PHQ-9	N/A	9.57 (5.15)	8.83 (4.85)
GAD-7	N/A	7.37 (3.93)	6.64 (3.53)
AUDIT-C	N/A	2.69 (2.33)	2.99 (2.52)
PSS-10	21.2 (2.78)	21.5 (4.18)	18.9 (4.98)
MAAS	57.5 (10.6)	60.2 (12.8)	64.1 (11.0)
CSQ-I	24.7 (6.14)	N/A	N/A
**WLC condition**			
Cannabis use past 30 days	22.5 (10.0)	22.0 (10.0)	22.0 (10.9)
Standard joints past 7 days	21.9 (24.7)	16.9 (13.4)	17.7 (20.8)
CUDIT	24.5 (7.37)	22.4 (7.53)	20.5 (9.09)
SDS	8.46 (4.00)	7.96 (3.92)	6.96 (3.88)
PHQ-9	N/A	10.3 (5.26)	9.84 (5.25)
GAD-7	N/A	7.99 (4.05)	7.07 (4.69)
AUDIT-C	N/A	2.99 (2.50)	2.70 (2.18)
PSS	21.4 (3.55)	21.3 (4.01)	20.7 (3.47)
MAAS	54.3 (13.6)	55.0 (11.8)	58.0 (13.7)
CSQ-I	N/A	N/A	N/A

AUDIT-C, Alcohol Use Disorders Identification Test; CSQ-I, Client Satisfaction Questionnaire; CUDIT, Cannabis Use Disorder Identification Test; GAD-7, General Anxiety Disorder; PHQ-9, Patient Health Questionnaire; SDS, Severity of Dependence Scale.

Regarding cannabis problems, both the CUDIT and SDS showed a significant Group × Time interaction (p < 0.001). At 6 months, both active interventions demonstrated substantial reductions compared to WLC, with no significant difference between them on the CUDIT. A sensitivity analysis on the SDS, however, revealed a significant difference favoring Mindfulness CANreduce (MD = −1.68, p = 0.010).

For perceived stress (PSS), both active groups improved significantly more than WLC, with no difference between them. For depressive symptoms (PHQ-9) and anxiety (GAD-7), however, significant interactions were found (p = 0.004 and p = 0.002, respectively), with Mindfulness CANreduce demonstrating superior outcomes over both WLC and Standard CANreduce at 6 months. For the MAAS, both active groups achieved higher mindfulness scores than WLC (p < 0.001). The AUDIT-C showed a significant group × time interaction (p = 0.043), whereby Mindfulness CANreduce showed higher scores than WLC in imputed analyses (MD = 0.57, p = 0.025), but not in complete-case analyses (MD = 0.15, 95% CI [−0.33, 0.64], p = 0.743).

### 3.5 Concurrent treatment utilization

Concurrent treatment use was minimal and did not differ between groups at 3 months (8.6%, p = 0.627) or 6 months (7.6%, p = 0.754), suggesting negligible contamination of treatment effects.

### 3.6 Client satisfaction

Client satisfaction (CSQ-I) at 6 weeks was high in both active groups (Mindfulness CANreduce M = 27.0, Standard CANreduce M = 24.7), with no significant difference in a Mann-Whitney *U* test used because of non-normality (Shapiro-Wilk, p < 0.001; U = 1346, p = 0.058). These scores stem only from participants retained at 6 weeks and may overestimate satisfaction among all randomized participants.

### 3.7 Adherence and retention rates

Server-log engagement data were available for the 174 of 203 active-arm participants (85.7%) registered on the Swiss platform; logs from the Austrian platform (n = 29) could not be retrieved. Participants who never accessed the intervention were scored as zero. Mean adherence (average completion across the eight modules) was 37.9% (SD = 32.7%; median 28.8%, IQR 10.9–62.0%), and 18% never accessed any module. Adherence did not differ significantly between the Standard (median 37.5%, IQR 12.5–62.5%) and Mindfulness (median 25.0%, IQR 9.4–57.0%) groups (Mann-Whitney *U* test, p = 0.18). Module completion declined monotonically from Module 1 to Module 8 in both arms (Supplementary Tables S2–S3).

Mean weekly diary retention in the same subsample was 33.4% (SD = 37.5%), with a median of 17.9% (IQR 0–71.4%). The Mindfulness CANreduce group had a descriptively higher retention rate (median 21.4%) than the Standard CANreduce group (median 11.9%), but the difference was not significant (Mann-Whitney *U* test, p = 0.33).

Use of the mindfulness-specific components could be quantified only in part. Among the 94 Mindfulness CANreduce participants with log data, 36 (38.3%) recorded at least one formal mindfulness practice in the consumption diary at any point during or after the 6-week programme (34, 36.2%, within the six intervention weeks); those who did logged a median of 5 practices (IQR 2–9) over a median of 4 days (IQR 2–7). Access to the audio-guided exercises was not captured by the server logs, so these figures are a lower bound of actual practice.

In an exploratory analysis (n = 56 with log data and observed 6-month outcomes), cannabis use days at 6 months were not significantly associated with module completion (IRR per 10 percentage points = 0.96, 95% CI [0.87, 1.05], p = 0.34), adjusted for baseline use and study arm. Participants who completed all eight modules reported the fewest use days (median 3, n = 12), but engagement strongly predicted completing the 6-month assessment itself (9% versus 67% among those finishing no versus all modules), so these estimates are susceptible to self-selection and should not be interpreted causally (Supplementary Table S4).

### 3.8 Negative effects

At the 6-week follow-up, 24 out of 156 participants (15.4%) reported at least one negative effect attributed to the programme, and 12 of them described the event. The reported events were heterogeneous and included psychological distress (e.g., increased anxiety, depressive phases), decreased motivation, and somatic complaints.

Of these 12 events, 7 (58.3%) had improved in severity from the time of occurrence to the time of reporting, and 5 (41.7%) were unchanged. No events were reported to have worsened over time.

## 4 Discussion

This three-arm RCT is the first direct comparison of a digital mindfulness-CBT intervention (Mindfulness CANreduce) against a CBT-only active comparator and a WLC for frequent cannabis users. Both active interventions reduced cannabis use days post-intervention, but only Mindfulness CANreduce demonstrated superior, sustained reductions at the 6-month follow-up. The mindfulness intervention also led to significantly greater improvements in depressive and anxiety symptoms than both the Standard CANreduce and WLC groups.

The short-term efficacy of both active interventions over the WLC aligns with meta-analytic evidence on digital interventions for cannabis reduction (Côté et al., 2024; Boumparis et al., 2019; Hoch et al., 2016). Both active arms outperformed the WLC at 6 weeks, and the newly developed Mindfulness CANreduce intervention matched its extensively refined predecessor at this time point.

However, the most critical finding is the difference in outcomes at 3 and 6 months. The advantage of the CBT-only Standard CANreduce intervention over the WLC diminished over time, becoming non-significant for the primary outcome from the 3-month follow-up onward. This reflects a common difficulty of digital interventions in achieving long-term results (Boumparis et al., 2019). By contrast, the Mindfulness CANreduce group maintained its initial gains and showed continued significant reductions in cannabis use between the 3- and 6-month assessments. This finding suggests that integrating mindfulness may help promote sustained behavioural change in digital cannabis interventions, although the underlying mechanisms and causal pathways remain to be established.

Several mechanisms may explain why mindfulness training may be particularly effective for cannabis users. Craving is experienced as a transient wave of sensations, emotions, and thoughts; urge surfing trains users to observe this wave with nonreactive, present-moment awareness until it subsides rather than responding with use (Bowen and Marlatt, 2009; Witkiewitz et al., 2005), while decentering and acceptance may weaken the link between negative affect and consumption (Witkiewitz and Bowen, 2010), consistent with momentary data linking state mindfulness to lower negative affect and cannabis desire (Shrier and Harris, 2023). Greater metacognitive awareness of triggers may additionally interrupt the largely automatic chain from cue to consumption (Breslin et al., 2002; Brewer et al., 2013). These skills plausibly build with continued practice, which may explain why advantages emerged most clearly at the later follow-ups.

One possible account of the sustained superiority of the Mindfulness CANreduce intervention is that mindfulness practice promoted generalized improvements in self-regulation, stress management, and emotional awareness extending beyond a reduction in cannabis use. The programme repeatedly encouraged audio-guided exercises, and regular practice may have fostered transferable skills, which could explain the continued improvement observed between the 3- and 6-month follow-ups. This account remains a hypothesis and must be reconciled with the low objective engagement observed (median module completion of 28.8% among participants with available log data). Module completion is, however, an imperfect proxy for skill practice: the audio-guided exercises could be repeated outside the module sequence and after the 6-week intervention period, the observed increase in trait mindfulness (MAAS) is consistent with acquired present-moment skills, and diary retention was descriptively higher in the Mindfulness arm. At the same time, only a minority of participants recorded formal mindfulness practices in the diary, so the extent of practice underlying these effects remains uncertain.

Mindfulness CANreduce also produced superior effects on the secondary mental health outcomes, with significant reductions in depressive (PHQ-9) and anxiety (GAD-7) symptoms at 6 months compared to both the standard CANreduce programme and the WLC. The hypothesised mindfulness mechanisms of decentring and acceptance may provide users with more effective strategies for managing the negative emotions that often trigger substance use. Building on prior evidence that CBT-based digital interventions show promise for improving the mental health of problematic cannabis users (Malischnig et al., 2020), the current findings suggest that explicit mindfulness training adds further benefit for common comorbidities.

The small increase in AUDIT-C scores in the Mindfulness CANreduce group relative to the WLC in the imputed analysis may reflect substance substitution, whereby reduced cannabis use is partially offset by increased alcohol consumption. However, the effect was small (MD = 0.57), was not present in the complete-case analysis (MD = 0.15, p = 0.74), and mean AUDIT-C scores remained in a low-risk range in all groups at all time points. Future digital cannabis trials should nevertheless monitor alcohol outcomes systematically.

Trait mindfulness (MAAS) also improved significantly in the Standard CANreduce arm, which contained no explicit mindfulness content. The CBT components focusing on self-monitoring, trigger awareness and craving management may cultivate present-moment awareness, regular tracking of cannabis use in the consumption diary may enhance metacognitive awareness, and successful behavioural change itself may increase general self-awareness. Structured self-help interventions may therefore promote mindfulness through multiple pathways.

The primary strength of this trial is its three-arm design with an active comparator, which enables a more rigorous evaluation of the mindfulness components, independent of the non-specific effects of receiving any digital intervention. The assessment up to six months addresses a key limitation of previous studies.

However, several limitations must be acknowledged. Firstly, only 31.6% of participants completed the final six-month follow-up. Attrition of this magnitude is common in fully automated digital intervention trials without human guidance (Eysenbach, 2005), but it raises legitimate concerns about selection bias, and the MAR assumption underlying the imputation model cannot be verified: dropout driven by unobserved factors such as relapse or dissatisfaction cannot be excluded. The observed patterns bound the plausible impact of attrition without establishing MAR. Dropout did not differ across arms at 6 months, gender was the only baseline predictor of missingness, the imputed and complete-case analyses converged on the primary 6-month contrasts, and the 6-month Mindfulness versus Standard contrast would be lost only under a severe, one-sided departure from MAR. Nevertheless, the divergence for the Standard CANreduce group at three months illustrates that missing-data assumptions can influence intermediate findings, and the 6-month results remain conditional on those assumptions. The absence of compensation at the 6-week assessment likely contributed. Retention remained low even though email reminders were supplemented by telephone contact. This pattern mirrors the anonymous, low-commitment use typical of digital self-help, and trials that require higher follow-up will likely need more intensive retention procedures, such as compensation at every assessment.

Secondly, the study recruited 301 of the initially planned 630 participants; despite an extended timeline, enrolment was concluded based on a realistic assessment of recruitment feasibility. The principal consequences are wider confidence intervals and an elevated risk of effect-size overestimation, as statistically significant effects detected in underpowered trials tend to overestimate true effects. Interpretation should therefore rest on the reported interval estimates rather than on statistical significance alone, and replication in an adequately powered confirmatory trial is warranted.

Thirdly, due to the nature of the interventions, it was not possible to blind either the participants or the researchers analysing the data, which introduces the potential for performance and detection bias. Outcomes were collected with validated self-report instruments, and we attempted to mitigate expectancy effects by informing those in the active groups that they were receiving a ‘revised’ version, but awareness of being on a waiting list can still influence outcomes. Importantly, the key 6-month difference emerged in the comparison least susceptible to such expectancy effects, Mindfulness versus Standard CANreduce, in which both groups received an active digital intervention.

Fourthly, exact age was available only for participants on the Swiss platform (n = 257). For the 44 participants on the Austrian platform, only the eligibility criterion of at least 18 years of age was verified, so the reported age distribution describes the Swiss subsample.

Fifthly, the exclusion of individuals reporting any suicidal ideation (26.5% of those screened) limits the generalisability of the findings. This criterion was necessary for participant safety in a fully automated intervention without clinical oversight, but the resulting sample may not represent the broader population of cannabis users seeking help. Given the high comorbidity of cannabis use disorders and suicidal ideation, future research should explore how to safely support individuals with suicidal thoughts.

Sixthly, the WLC condition was not a no-treatment condition but reflected internet as usual: waitlisted participants retained free access to other preventive and therapeutic services, and in previous CANreduce trials isolated waitlisted participants registered in parallel with a comparable German-language digital programme. Such co-intervention biases against the active arms, making the reported comparisons conservative. The condition mirrors the predecessor trial's control (Baumgartner et al., 2021), supporting comparability across the CANreduce programme, and withholding a complete intervention from help-seeking users beyond a delay would have been ethically problematic. WLC participants received access only after the final 6-month assessment, so delayed access cannot have contaminated the primary comparisons; concurrent treatment utilization was low and comparable across groups.

Seventhly, the two active interventions were not matched for time and attention: the Mindfulness CANreduce arm added audio-guided exercises, practice recommendations, and practice tracking to a CBT core of comparable text length, so its superiority may partly reflect greater intervention exposure rather than mindfulness content itself. A dismantling design with an attention-matched comparator is needed to isolate the specific contribution of mindfulness practice.

Eighthly, all outcomes were self-reported and cannabis use was not biochemically verified. Engagement could be characterized only partially: server logs were unavailable for the Austrian platform, and use of the audio-guided mindfulness exercises was not logged, so the extent of actual mindfulness practice remains uncertain.

Finally, the low adherence and retention rates suggest that participants, on average, engaged with only a minority of the intervention content (median module completion of 28.8% among those with available log data). While this indicates that the observed effects were achieved with low engagement, which is relevant for real-world implementation, it also emphasises the need for better engagement strategies in future iterations.

In this trial, a digital self-help intervention integrating mindfulness and CBT was associated with more sustained reductions in cannabis use and greater improvements in depressive and anxiety symptoms than a CBT-only approach in frequent cannabis users, although the 6-month findings are conditional on assumptions about the missing data. These findings suggest that mindfulness skills may help individuals manage cravings and negative emotions beyond the short term. Despite significant challenges with high dropout rates and low engagement, the Mindfulness CANreduce programme represents a promising and scalable approach to the public health burden of cannabis use disorder.

## CRediT authorship contribution statement

N.B. led the formal analysis, visualization, and original draft writing. M.P.S. led the conceptualization, funding acquisition, methodology, and supervision. M.D. led the investigation. A.W. led data curation and software resources. D.M. contributed to the original draft and conceptualization. C.B. contributed to software and methodology. S.H. contributed to conceptualization. All authors reviewed and approved the final manuscript.

## Ethical approval and consent to participate

The study protocol was approved by the Ethics Committee of the Faculty of Arts and Social Science at the University of Zurich, Switzerland (approval number: 20.4.17). All participants gave their informed consent before starting the intervention.

## Trial registration

International Standard Randomized Controlled Trial Number Registry: ISRCTN14971662; Date of registration: 09/09/2021.

## Funding

This work was supported by the 10.13039/501100001711Swiss National Science Foundation (SNSF; grant number 10001C_192301). The SNSF had no role in study design, data collection, analysis, or interpretation, or in manuscript preparation.

## Declaration of competing interest

The authors declare no competing financial or non-financial interests.

## Data Availability

The datasets generated and analysed during the current study are not publicly available due to privacy considerations inherent in clinical trial data containing sensitive health information. However, they are available from the corresponding author upon reasonable request. Requests will be reviewed within 30 days, and data access agreements will be required to ensure the data is handled appropriately.
