# Supplementary material for: Added value of mindfulness in a digital cannabis intervention: Three-arm randomized trial
Source: Internet Interv. 2026 Nov 3;46:101021. doi: 10.1016/j.invent.2026.101021 (PMC13756303; doi:10.1016/j.invent.2026.101021)
Supplement: Supplementary file 1 [file mmc1.docx]

**Supplementary Material**

**Delta-adjusted (tipping-point) sensitivity analysis of the 6-month primary outcome**

**Rationale**

The primary analysis handled missing outcome data through multiple imputation by chained equations under the Missing At Random (MAR) assumption. Because MAR cannot be verified empirically, and because dropout in addiction trials may be driven by unobserved post-baseline factors such as relapse or dissatisfaction with the intervention, we conducted a delta-adjusted (tipping-point) sensitivity analysis. This analysis quantifies how strongly the unobserved 6-month outcomes of participants lost to follow-up would need to depart from their MAR-based imputed values before the key 6-month conclusions change. If the departure required to overturn a finding is implausibly large, the finding can be considered robust to violations of MAR.

**Methods**

At the 6-month follow-up (FU2), the primary outcome (cannabis use days in the preceding 30 days) was missing for 78 of 109 participants (71.6%) in the Mindfulness CANreduce arm, 63 of 94 (67.0%) in the Standard CANreduce arm, and 61 of 98 (62.2%) in the waitlist control (WLC) arm. Under MAR-based imputation, the mean imputed FU2 value among Mindfulness-arm participants with missing data was 8.76 use days (their baseline mean: 25.55 days; the observed mean among Mindfulness-arm completers: 11.06 days).

The sensitivity parameter delta (δ) represents additional cannabis use days per month among Mindfulness-arm dropouts beyond what MAR-based imputation predicts. For each δ from 0 to 17, we added δ to the imputed FU2 values of Mindfulness CANreduce participants with missing 6-month data only, truncated values to the admissible range of 0-30 days, and refitted the exact primary model: a zero-inflated negative binomial generalized linear mixed model (glmmTMB) with fixed effects for group, time, and their interaction, the standardized baseline value as covariate, a random intercept per participant, and a zero-inflation component depending on time. Pairwise group contrasts at FU2 were computed from estimated marginal means with Tukey adjustment, identical to the primary analysis. Imputed values in the comparator arms were left unchanged; the analysis therefore represents a deliberately conservative, one-directional stress test in which only the intervention arm is penalized.

At δ = 0 the analysis exactly reproduces the published 6-month estimates (Mindfulness vs Standard: IRR = 0.52, 95% CI [0.43, 0.64]; Mindfulness vs WLC: IRR = 0.46, 95% CI [0.38, 0.56]; Standard vs WLC: IRR = 0.88, p = .244), confirming the fidelity of the implementation.

**Results**

Table S1 reports the two Mindfulness contrasts at FU2 for each δ. Figure S1 displays the IRRs with 95% confidence intervals.

**Table S1. Delta-adjusted 6-month contrasts (Tukey-adjusted).**

| δ (days) | Mindf vs Standard IRR | p | Mindf vs WLC IRR | p |
| --- | --- | --- | --- | --- |
| 0 | 0.524 | <.0001 | 0.461 | <.0001 |
| 1 | 0.498 | <.0001 | 0.438 | <.0001 |
| 2 | 0.533 | <.0001 | 0.469 | <.0001 |
| 3 | 0.568 | <.0001 | 0.500 | <.0001 |
| 4 | 0.602 | <.0001 | 0.530 | <.0001 |
| 5 | 0.636 | <.0001 | 0.559 | <.0001 |
| 6 | 0.669 | <.0001 | 0.588 | <.0001 |
| 7 | 0.701 | <.0001 | 0.616 | <.0001 |
| 8 | 0.733 | .0001 | 0.644 | <.0001 |
| 9 | 0.765 | .0010 | 0.672 | <.0001 |
| 10 | 0.797 | .0063 | 0.699 | <.0001 |
| 11 | 0.829 | .0294 | 0.727 | <.0001 |
| **12** | **0.861** | **.1017** | 0.754 | .0001 |
| 13 | 0.893 | .2642 | 0.782 | .0006 |
| 14 | 0.924 | .5242 | 0.809 | .0039 |
| 15 | 0.956 | .8075 | 0.836 | .0184 |
| **16** | 0.987 | .9832 | **0.863** | **.0658** |
| 17 | 1.019 | .9635 | 0.890 | .1810 |

Bold rows mark the tipping points (first δ at which the Tukey-adjusted p ≥ .05). The Standard vs WLC contrast remained non-significant across the entire grid (p = .16-.26). The slight non-monotonicity between δ = 0 and δ = 2 reflects the interplay between the shifted count values and the zero-inflation component of the model.

**Interpretation**

The 6-month Mindfulness versus Standard CANreduce contrast loses statistical significance only at δ = 12, that is, only if every Mindfulness-arm participant lost to follow-up used cannabis on average 12 days per month more than the MAR-based imputation predicts. This corresponds to a mean of 18.9 use days in this subgroup, which is close to their baseline average of 25.6 days and well above the average of 11.1 days observed among Mindfulness-arm completers. It additionally requires that dropouts in the Standard and WLC arms simultaneously behaved exactly as MAR predicts, an asymmetry for which there is no empirical indication: attrition was non-differential across arms, and baseline characteristics did not differ between completers and non-completers.

The Mindfulness versus WLC contrast is even more robust, tipping only at δ = 16 (a mean of 22.2 use days, a near-complete return to baseline). We therefore conclude that the 6-month superiority of the Mindfulness CANreduce intervention is robust to all but extreme and one-sided violations of the MAR assumption.

**Figure S1. Delta-adjusted tipping-point sensitivity analysis of the 6-month primary outcome.**

**Intervention engagement analyses**

**Data source and definitions**

Engagement was quantified from server logs of the CANreduce 3.0 platform (page-view records per module and weekly consumption diary records). Logs were available only for participants registered on the Swiss platform: 174 of the 203 randomized active-arm participants (85.7%; Mindfulness CANreduce n = 94, Standard CANreduce n = 80). No log exports exist for the 29 active-arm participants registered on the Austrian platform; engagement analyses are therefore restricted to the Swiss subsample. Module completion was computed per module as the highest page reached divided by the number of pages in the module, adherence as the mean completion across all eight modules, and weekly diary retention as the number of diary days completed divided by seven, averaged across the six intervention weeks. Participants who never accessed the intervention were scored as zero on all engagement metrics.

**Table S2. Engagement descriptives by study arm (Swiss platform subsample).**

| **Metric** | **All active arms (n=174)** | **Mindfulness CANreduce (n=94)** | **Standard CANreduce (n=80)** | **Group comparison** |
| --- | --- | --- | --- | --- |
| Adherence, mean (SD) | 37.9% (32.7) | 35.8% (33.9) | 40.3% (31.3) | Mann-Whitney U, p = 0.18 |
| Adherence, median (IQR) | 28.8% (10.9-62.0) | 25.0% (9.4-57.0) | 37.5% (12.5-62.5) |  |
| Never accessed any module, n (%) | 31 (17.8%) | 19 (20.2%) | 12 (15.0%) |  |
| Diary retention, mean (SD) | 33.4% (37.5) | 36.1% (37.5) | 30.1% (37.5) | Mann-Whitney U, p = 0.33 |
| Diary retention, median (IQR) | 17.9% (0-71.4) | 21.4% (0-73.8) | 11.9% (0-46.4) |  |

**Table S3. Module-level access and completion by study arm (Swiss platform subsample).**

| **Module** | **Mindfulness: accessed** | **Mindfulness: fully completed** | **Mindfulness: mean progress** | **Standard: accessed** | **Standard: fully completed** | **Standard: mean progress** |
| --- | --- | --- | --- | --- | --- | --- |
| 1 | 79% | 66% | 75% | 82% | 79% | 80% |
| 2 | 61% | 52% | 57% | 69% | 68% | 69% |
| 3 | 44% | 41% | 42% | 62% | 54% | 57% |
| 4 | 38% | 33% | 36% | 44% | 39% | 41% |
| 5 | 30% | 24% | 27% | 34% | 28% | 30% |
| 6 | 22% | 16% | 19% | 25% | 18% | 20% |
| 7 | 17% | 14% | 15% | 15% | 14% | 14% |
| 8 | 16% | 14% | 15% | 12% | 11% | 12% |

Accessed: viewed at least one page of the module. Fully completed: reached at least 99% of the module pages.

**Mindfulness practice diary (Mindfulness CANreduce arm)**

In the Mindfulness CANreduce arm, participants could log formal mindfulness practices in the consumption diary. Among the 94 participants with available log data, 36 (38.3%) logged at least one practice at any point during or after the 6-week programme, and 34 (36.2%) did so within the six intervention weeks. Among these 36 participants, the median number of logged practices was 5 (IQR 2-9; maximum 132), distributed over a median of 4 practice days (IQR 2-7) and a median of 2 distinct weeks (IQR 1-3).

**Exploratory analysis of engagement and 6-month outcome**

Among participants with both log data and an observed 6-month primary outcome (n = 56; 28 per active arm), cannabis use days at 6 months were regressed on adherence (per 10 percentage points of module completion), standardized baseline use days, and study arm, using a negative binomial model on observed data. This analysis is exploratory and cannot separate the effect of engagement from self-selection.

**Table S4. Exploratory engagement-outcome analysis.**

| **Predictor** | **IRR** | **95% CI** | **p** |
| --- | --- | --- | --- |
| Adherence (per 10 percentage points) | 0.96 | 0.87-1.05 | 0.34 |
| Baseline use days (z) | 1.53 | 1.13-2.07 | 0.006 |
| Arm (Standard vs Mindfulness) | 1.39 | 0.78-2.48 | 0.27 |

Descriptively, mean cannabis use days at 6 months varied across engagement groups, defined by the number of fully completed modules. Mean use was 10.2 days among participants completing no modules, 16.2 days among those completing 1–3 or 4–7 modules, and 8.3 days among those completing all 8 modules. The corresponding medians were 4, 20, 12, and 3 days, respectively. Six-month assessment completion increased monotonically across engagement groups, from 9% among participants completing no modules to 29%, 48%, and 67% among those completing 1–3, 4–7, and all 8 modules, respectively. This pattern suggests that the observed outcomes at 6 months may be subject to selection bias, as participants with higher engagement were more likely to complete the follow-up assessment. Accordingly, these descriptive findings should not be interpreted as evidence of a causal dose-response relationship.

**Quantification of cannabis quantity**

Cannabis quantity over the past 7 days was assessed with an online-adapted Timeline Follow-Back method. For quantification purposes, participants first defined their personal standard joint by selecting cannabis type (indoor, outdoor, or resin), tobacco admixture status (with or without), and quantity from six predetermined amounts ranging from 67 to 500 mg. This personalized standardization procedure yielded 36 possible combinations, each visually represented through photographs of unrolled joint papers displaying the selected cannabis (and tobacco) quantities alongside a ruler for scale reference. This individually calibrated standard joint subsequently served as the unit of measurement for self-reported cannabis consumption.

**Figure S2.** Standard-joint definition screen of the baseline assessment. Participants chose the cannabis product they usually consume (outdoor cannabis, indoor cannabis, or resin), whether they usually mix it with tobacco, and the amount of cannabis (67, 100, 167, 250, 333, or 500 mg). An image of an unrolled joint paper next to a ruler shows the selected quantity. Participants who mainly vaporise were asked to report joint equivalents, and CBD joints were not counted. The choice was final and also set the unit of the consumption diary.
